# The smallest things make me emotional! Emotion reactivity in non-suicidal self-injury: trait, state, and physiological differences

**DOI:** 10.3389/fpsyg.2024.1309187

**Published:** 2024-08-23

**Authors:** Melissa S. Jankowski, Aubrey J. Legasse, Victoria Marques, Meaghan L. Delcourt, Emily A. P. Haigh

**Affiliations:** ^1^Department of Psychology, University of Maine, Orono, ME, United States; ^2^State Forensic Service, Augusta, ME, United States; ^3^Lifespan, Newport, RI, United States; ^4^Department of Psychology, University of Connecticut, Storrs, CT, United States; ^5^Department of Psychology, University of Victoria, Victoria, BC, Canada

**Keywords:** non-suicidal self-injury, emotion reactivity, physiological measurement, state-trait measurement, Cyberball

## Abstract

**Objective:**

The current study sought to clarify the role of emotion reactivity in non-suicidal self-injury (NSSI) by examining three forms of emotion reactivity (physiological and self-reported trait and state reactivity) among a sample of young adults with or without a history of NSSI.

**Materials and methods:**

Seventy-six adults (*M*_age_ = 20.97, 73.7% female) participated in a semi-structured clinical interview to determine NSSI history and completed a measure of trait emotion reactivity. Participants then provided state emotion reactivity ratings before and after a social rejection task, recovery period, and positive mood induction while physiological data was continuously recorded.

**Results:**

Although individuals with a history of NSSI perceived themselves to be more emotionally reactive, these participants were not more physiologically reactive, nor were their state reactivity ratings significantly different from individuals without a history of NSSI.

**Discussion:**

Results suggest increased emotionality in response to a stressor is within normal bounds and not unique to individuals with a history of NSSI, and provide implications for future research on the etiology and treatment of NSSI.

## Introduction

Non-suicidal self-injury (NSSI; i.e., deliberately harming oneself without the intent to die) is a significant public health concern with lifetime prevalence of NSSI around 17% for adolescents, 13% for young adults, and 5.5% for adults (Swannell et al., 2014). Research synthesizing NSSI prevalence indicates that rates of NSSI are increasing (see Cipriano et al., 2017 for review). This rise in prevalence is particularly alarming given the evidence that NSSI is associated with future suicide ideation and attempts (You and Lin, 2015; Castellví et al., 2017; Koenig et al., 2017), psychological symptoms (e.g., depression, anxiety, and borderline personality disorder; Lundh et al., 2011; Baetens et al., 2014), and cognitive vulnerability to future psychological distress (Guerry and Prinstein, 2010; Garisch and Wilson, 2015). Theoretically grounded research is needed to understand factors that contribute to the intervention and prevention of NSSI.

Prominent theories of NSSI highlight the putative role of emotion dysregulation, or the inability to flexibly respond to and manage emotions (Nock and Prinstein, 2004; Chapman et al., 2006; Nock, 2009, 2010; Werner and Gross, 2010). Emotion dysregulation reflects impairment in emotion regulation (i.e., difficulty modulating emotional states; Gratz and Roemer, 2004) and emotion reactivity (i.e., the threshold, intensity, and duration of experienced emotions; Davidson, 1998). It is theorized that individuals who engage in NSSI experience high levels of negative affect that they are unable to moderate, which renders them hyperreactive to emotional stimuli. By providing emotional relief, NSSI is reinforced as an effective, albeit maladaptive emotion regulation strategy (e.g., Selby and Joiner, 2009; Hasking et al., 2016). In short, emotion reactivity may predispose individuals to difficulties in emotion regulation, which may in turn increase risk for NSSI and suicide (Nock and Mendes, 2008).

In response to mixed evidence, researchers have begun to question whether emotion reactivity plays a direct and central role (Hooley and Franklin, 2017). Specifically, it is unclear whether individuals who engage in NSSI actually do so in response to extreme episodes of intense, prolonged, negative affect (i.e., emotion reactivity). Evidence supporting the role of emotion reactivity in NSSI is mixed, leading researchers to question whether emotion reactivity plays a direct and central role (Hooley and Franklin, 2017). It is possible that an accurate understanding of the role of emotion reactivity in NSSI is being obscured by common-method variance. A multimethod approach is needed to better understand whether individuals with or without a history of NSSI differ in their experience of emotion reactivity, and, if so, how these groups differ in their experience (Zelkowitz et al., 2016). The current study examines three forms of emotion reactivity in response to a mood induction paradigm: trait, state changes in affect, and physiological reactivity. We acknowledge that our state and trait measures represent subjective self-reports and our state and physiological measures both reflect moment to moment changes in emotion reactivity. For the purposes of this study, we will refer to these overlapping constructs as trait, state, and physiological emotion reactivity.

### Background literature

Emotion reactivity among individuals with or without NSSI is most commonly studied using self-report trait measures that asks an individual to assess how they typically experience emotions on a regular basis. While evidence from cross-sectional studies have found large differences in self-reported trait emotion reactivity when comparing NSSI to non-NSSI groups (e.g., Nock and Mendes, 2008; Glenn and Klonsky, 2011; Andover and Morris, 2014; Zelkowitz et al., 2016; Mettler et al., 2023), with NSSI groups endorsing higher scores. In contrast, findings from daily diary studies have generally failed to reveal differences in self-reported trait emotion reactivity (Bresin, 2014), with the exception of those studies that include participants with symptoms of borderline personality disorder (Houben et al., 2017; Santangelo et al., 2017). Given these mixed results, and the fact that trait measures are not designed to measure emotions as they unfold in real time, research is needed to test whether trait measurement of emotion reactivity is similar to state measures, such as self-report and physiological measures.

To explore state changes in emotion reactivity, quasi-experimental studies have explored self-reported state emotion reactivity in response to mood induction paradigms. Similar to trait emotion reactivity research, results from quasi-experimental research have demonstrated mixed findings. For instance, a small number of quasi-experimental studies have found participants with NSSI history report higher levels of negative affect at baseline, but not in response to a negative, positive, or angry mood induction (Weinberg and Klonsky, 2012; Mettler et al., 2021) or a stressful speech task (Franklin et al., 2010; Kaess et al., 2012). Other researchers have found that individuals with a history of NSSI exhibited *less* negative affect in response to a negative mood induction (Bresin and Gordon, 2013; Boyes et al., 2020) and *less* positive affect following a positive mood induction than individuals without NSSI history (Boyes et al., 2020). Conversely, individuals with a history of NSSI reported increased negative and decreased positive affect following a rumination induction compared to individuals with a history of eating disorder behaviors (Arbuthnott et al., 2015). Given the small number of mood induction studies and inconsistent results, additional research is needed to examine changes in mood-induced affect among individuals with a history of NSSI as they occur in real time. Such an investigation would allow for researchers to observe group differences in the persistence, duration, and threshold of these emotional reactions in response to different mood states. Further, longitudinal research has been conducted to explore the relationship between emotional reactions and NSSI over time. Like the mixed results of quasi-experimental studies suggest, longitudinal studies call into question the central role of emotion reactivity in predicting future NSSI. For example, results of a meta-analysis found that while affect dysregulation predicted NSSI, the odds ratio was weak (i.e., 1.05) and the authors concluded most longitudinal studies have not measured emotion reactivity, but rather assessed constructs such as depressive and anxious symptoms (Fox et al., 2015).

To better understand the physiological concomitants of subjective measures of state and trait emotion reactivity, research has begun to examine the role of high-frequency heart rate variability (HF-HRV), a biomarker for self-regulation and an index of respiratory sinus arrhythmia (RSA; variation in heart rate across the respiration cycle) within the context of NSSI (Beauchaine and Thayer, 2015). HF-HRV is the variability of interbeat intervals between consecutive heartbeats and indicates central regulation of the autonomic nervous system, which can be used as a measure of parasympathetic control and vagal tone (Berntson et al., 1997; Porges, 2007). Polyvagal Theory (Porges, 2007) holds that high parasympathetic nervous system-mediated (PNS-mediated) activity in a neutral state is protective and adaptive; however, suppression of PNS-mediated activity, or withdrawal of the “vagal brake,” during stress is indicative of flexible mobilization to environmental stressors, enabling the fight or flight response. Thus, while higher resting-state HF-HRV is adaptive, increased HF-HRV withdrawal in response to stress is thought to relate to dysregulation in emotional coping (Appelhans and Luecken, 2006; Rottenberg, 2007). Aberrant patterns of HF-HRV have been associated with risk for a number of psychopathological symptoms, including anxiety, borderline personality disorder, depression, and self-injurious thoughts and behaviors (Crowell et al., 2005; Beauchaine and Thayer, 2015; Wilson et al., 2016). As HF-HRV has been utilized as a biomarker for self-regulation, its relationship with emotion regulation is of particular importance, especially in the context of NSSI. Emotion dysregulation is consistently associated with dysregulated PNS- and SNS-mediated biological reactivity throughout different psychopathologies and includes both cardiovascular and neuroendocrine measures (Ingjaldsson et al., 2003; Quartana and Burns, 2010; Denson et al., 2011; DiSimplicio et al., 2012; Beauchaine and Thayer, 2015).

Efforts to examine individual differences in physiological indices of emotion reactivity have yielded mixed results. Crowell et al. (2005) found adolescents who engaged in self-injurious behavior exhibited greater RSA withdrawal, specifically HF-HRV, at baseline and in response to a sad mood induction. Other research found no differences in heart rate or heart rate variability among adolescent females with or without a history of NSSI at resting-state (Koenig et al., 2017). Fox et al. (2018) examined resting-state, and stress-mediated RSA among a sample of 70 young adults with or without a recent history of NSSI. Though resting-state RSA did not predict a history of NSSI engagement, greater RSA withdrawal in response to a social stress task predicted recent NSSI. Future research is needed to clarify the role of HF-HRV at rest and in response to various types of affective challenge paradigms to explore emotion reactivity *in vivo*.

To our knowledge, no single study has examined whether individuals with or without a history of NSSI differ in trait, state, and physiological emotion reactivity. Using both state and trait indices may clarify whether individuals with NSSI history subjectively differ in the sensitivity and duration of their affective responses as compared to a comparison group. As NSSI is often influenced by affective states (e.g., Nock and Mendes, 2008; Hasking et al., 2016), manipulating affect, such as positive and negative mood, through various mood inductions, like a social ostracism task, recovery period, and positive mood induction, would help determine the role of emotion reactivity in NSSI and whether this should continue to be considered as an important point for intervention. Given that social ostracism (i.e., rejection) is particularly salient for young adults and is thought to precede NSSI in general (e.g., Selby et al., 2012; van Geel et al., 2015), Cyberball, a social ostracism task, was hypothesized to serve as a particularly salient negative mood induction in the current study. Similarly, HF-HRV has been shown to be particularly sensitive to social rejection (Cosley et al., 2010; Porges, 2011; Liddell and Courtney, 2018; Kroll et al., 2019). Thus, the current study sought to clarify how trait and mood-mediated state and physiological measures of emotion reactivity relate to NSSI group membership.

### The current study

To help clarify mixed findings regarding the role of emotion reactivity in NSSI, we examined whether individuals with a history of NSSI (NSSI group) differ in trait, state, and physiological emotion reactivity in response to a social ostracism task, recovery period, and positive mood induction. Data were drawn from a sample of adults who participated in a semi-structured clinical interview to determine NSSI history. Participants with or without a history of NSSI completed a measure of trait emotion reactivity, and then provided state affect ratings following a social ostracism task, recovery period, and positive mood induction, while HF-HRV data was continuously recorded.

A social ostracism task was chosen, as research has shown that NSSI behavior is associated with feelings of peer rejection (see Plener et al., 2015 for a review; Esposito et al., 2019). Peer rejection has been linked to various adverse outcomes, and evidence shows that peer rejection is a risk factor for NSSI in both adolescence and young adulthood (Fisher et al., 2012; Lereya et al., 2013; Plener et al., 2015 for a review; Kyron et al., 2018; Victor et al., 2019). To target this particular vulnerability, the current study employed Cyberball (Williams and Jarvis, 2006), an analog social ostracism task.

### Aims and hypotheses

First, this investigation sought to replicate studies that have found large differences in self-reported, trait levels of emotion reactivity in individuals who engage in NSSI compared to those who do not have a history of these behaviors (e.g., Nock et al., 2008; Glenn et al., 2011; Franklin et al., 2013). Compared to individuals without a history of NSSI, we predicted that participants with a lifetime history of NSSI would report higher baseline levels of trait emotion reactivity as measured by The Emotion Reactivity Scale (ERS) (Nock et al., 2008).

Next, we sought to examine whether individuals with a history of NSSI differed from individuals without NSSI history on state emotion reactivity in response to a social ostracism task. Given evidence that NSSI is often associated with feelings of rejection, we hypothesized that the NSSI group would show significantly greater changes in state emotion reactivity (i.e., increased negative affect) following the social ostracism task, the exclusion period of Cyberball.

Third, in line with previous findings that individuals who engage in NSSI demonstrate changes in physiological reactivity (e.g., Kelly et al., 2012; Sleegers et al., 2016), we predicted that the NSSI group in our sample would demonstrate significantly greater physiological arousal (i.e., increased withdrawal of HF-HRV) following the social ostracism task (i.e., Cyberball) than the comparison group.

Fourth, in response to the recovery period, and fifth, in response to the positive mood induction, we predicted that the NSSI group would demonstrate persistent physiological reactivity (i.e., increased HF-HRV withdrawal) and emotional persistence (i.e., less improvement in negative affect and less positive affect) compared to the comparison group. These hypotheses are in line with Boyes et al. (2020), who found that individuals with a history of NSSI in response to a negative and positive mood induction demonstrated persistent negative emotion over time and less positive emotion reactivity, respectively.

## Materials and methods

### Participants

The University’s Research Ethics Review Board approved the study prior to data collection. Participants were 76 adults (37 NSSI, 39 non-NSSI) recruited from a large university and surrounding community in the northeast region of the United States using convenience sampling. The mean age was 20.97 (range = 18–63), and participants were 73.7% female. A majority of participants identified as White (86.8%) and non-Hispanic or Latino (96.1%), with 5.3% identifying as Native American or Alaskan Native, 3.9% as Asian, 2.6% as Black or African American, and 1.3% as mixed or multiple races.

An *a priori* power analysis conducted using G*Power 3.1 (Faul et al., 2007) revealed a total sample size of 24 would result in an 80% chance of detecting a medium effect; thus, this study was sufficiently powered.

### Measures

#### Demographics

Participants reported demographic information, including age, gender identity, and racial and ethnic identities.

#### Depressive symptoms

Depressive symptoms were measured using the Beck Depression Inventory-Second Edition (BDI-II) (Beck et al., 1996), a 21-item self-report questionnaire that measures depressive symptomatology experienced during the previous 2 weeks. The internal consistency of the BDI-II was excellent in this study (*α* = 0.94).

#### Self-injurious thoughts and behaviors

NSSI was assessed using the Self-Injurious Thoughts and Behaviors Interview (SITBI) (Nock et al., 2007), a semi-structured interview designed to assess the presence, frequency, and characteristics of various self-injurious behaviors.

#### Trait emotion reactivity

Participants’ perceived trait emotion reactivity was measured using the Emotion Reactivity Scale (ERS) (Nock et al., 2008), a 21-item self-report measure of emotion reactivity that looks specifically at the perceived sensitivity, intensity, and persistence of emotions. Internal consistency for the present study was excellent (*α* = 0.96).

#### State emotion reactivity

##### Visual analog scale

The Visual Analog Scale (VAS) is a measure used to assess current mood and was used as a manipulation check. Participants were presented with a 100-mm line with “sad” and “happy” at the 0- and 100-mm points, and were asked to mark the numerical value that represented their present mood. Research has demonstrated that the VAS has adequate test–retest reliability and concurrent validity (Folstein and Luria, 1973; Little and McPhail, 1973).

##### Positive and negative affect schedule

The Positive and Negative Affect Schedule (PANAS) (Watson et al., 1988) is a 20-item self-report measure that assesses positive and negative affect. Participants rate 20 affective adjectives rated on a Likert Scale ranging from 1 to 5 (1 = *very slightly or not at all* to 5 = *extremely*). Research has shown adequate internal consistency for both scales at various time points and has demonstrated convergent and discriminant validity (Watson et al., 1988).

#### Physiological reactivity

##### High-frequency heart rate variability

High-frequency heart rate variability (HF-HRV) is a widely accepted measure of parasympathetic function (Berntson et al., 2007), and was used as a physiological measure of state emotion reactivity in this study. HRV data was collected and amplified with Mindware hardware and Biolab 3.1 (2009) acquisition software at a sampling rate of 1,000 Hz. Biolab software was utilised to clean and calculate HF-HRV/RSA data and parameters. Final HF-HRV/RSA data was derived utilizing Mindware’s HRV module following manual artifact editing of the digital recording of inter-beat intervals. A Fast Fourier Transform was used to derive HF-HRV frequency band distribution, typically within 0.15 and 0.4 Hz. Due to the inclusion of impedance cardiography, the impact of respiration rate was assessed to generate HF-HRV/RSA values.

##### Social ostracism task

Cyberball is a computerized, simulated social ostracism task in which participants think they are playing an online ball-toss game against two individuals. In reality, they are playing with programmed opponents (Williams and Jarvis, 2006). For the first third of the game, which lasts approximately 2 min, participants were included in game play (i.e., the first 40 throws). Following the inclusion period, participants were excluded from the last two-thirds of the game (i.e., remaining 80 throws) with no warning, which is about 2 min in length. This task reliably produces feelings of social stress and ostracism (see Hartgerink et al., 2015).

### Procedure

After participants provided informed consent, a graduate assistant or trained research assistant administered the SITBI (Nock et al., 2007) to assess the presence or absence of a lifetime history of NSSI. Next, non-invasive disposable sensors were attached to participants’ right collarbone, bottom left rib, bottom right rib, jugular notch, and sternum to record physiological data during the remainder of the study, which included the following tasks: baseline (7 min nature video), Cyberball (Williams and Jarvis, 2006), recovery period (7 min nature video), and a positive mood induction (3 min humorous video about Maru the Cat). Positive and negative affect were assessed after each task.

### Data analysis

Data was first inspected for missing data. Only the PANAS contained missing data, which was imputed using multiple imputation (Little and Rubin, 2002). HRV/RSA data for 14 participants (six NSSI, eight comparison group) were missing or unusable (e.g., too much participant movement, inaccurate sensor placement) and were excluded from physiological reactivity analyses.

Primary study variables were then examined for normality and the presence of outliers (Tabachnick and Fidell, 2007). Preliminary analyses were conducted to inspect whether the NSSI and comparison groups differed significantly on any demographic variables. State changes in self-reported emotion reactivity were calculated by difference scores (e.g., post-task PANAS minus pre-task PANAS) for Cyberball, recovery, and positive mood induction. A similar approach was used to calculate physiological reactivity. Baseline HF-HRV/RSA was computed by averaging the last 3 min of HF-HRV/RSA during the baseline period. The exclusion period was calculated by averaging the 2-min period when the participant was excluded in Cyberball. Reactivity scores were then calculated by looking at the differences between baseline and exclusion. Recovery was calculated by baseline minus the last 3 min of the recovery video. State emotion reactivity was measured on the PANAS following the baseline video, Cyberball, recovery video, and positive mood induction.

## Results

### Preliminary analyses

The NSSI group did not differ from the non-NSSI group on age, gender, or race (see Table 1). They did differ significantly in regard to current depressive symptoms, with the NSSI group reporting higher current depressive symptoms as measured by the BDI-II [*t*(53.78) = −7.44, *p* < 0.001]. Among the 37 participants in the NSSI group, approximately half reported having engaged in NSSI in the past year (*N* = 19, 51%), nine in the past month (24%) and six in the past week (16%). Reported lifetime episodes ranged from one to 1,000. The average age of onset was 14.09 years (range 6–21) and the most common methods reported were “cut or carved skin” (73%), “picked at a wound” (62%), “hit yourself on purpose” (54%), and “scraped your skin” (51%).

**Table 1:** Demographics of participant groups.

Variable	NSSI (*n* = 37)	Control (*n* = 39)	Range	Statistic
Mean (*SD*) age in years	20.2 (2.6)	21.7 (7.8)	18–63	*t*(73) = 1.02, *p* = 0.310
Gender (% female)	67.6	79.5		*X*^2^(1) = 1.391
Race/Ethnicity (%)				*X*^2^(1) = 0.348
Caucasian	89.2	84.6		
Black/African American	0.0	5.1		
Asian	8.1	0.0		
Native American	2.7	7.7		

NSSI, non-suicidal self-injury.

### Manipulation check

To ensure Cyberball was effective in eliciting a stress-mediated response, paired-samples *t*-tests were performed to examine whether there was a significant change in VAS scores before and after the task for the entire sample. VAS scores significantly decreased after Cyberball with medium to large effect sizes; *t*(74) = 6.05, *p* < 0.001, Cohen’s *d* = 0.70, which suggests Cyberball effectively elicited emotional responses for all participants, regardless of group.

### Trait emotion reactivity

A series of independent samples t-tests revealed the groups significantly differed on the ERS total score as well as all three subscales. Specifically, the NSSI group had higher total scores (*M* = 43.03, *SD* = 18.86) than the non-NSSI group [(*M* = 20.85, *SD* = 14.71); *t*(73) = −5.71, *p* < 0.001, Cohen’s *d* = 1.32]. The NSSI group (*M* = 18.83, *SD* = 9.13) also scored higher on the sensitivity subscale than the comparison group [(*M* = 9.03, *SD* = 7.33); *t*(73) = −5.15, *p* < 0.001, Cohen’s *d* = 1.19]. Similarly, on the arousal subscale, the NSSI group (*M* = 12.19, *SD* = 5.84) scored higher than the comparison group [(*M* = 5.77, *SD* = 4.31); *t*(73) = −5.45, *p* < 0.001, Cohen’s *d* = 1.25]. Finally, the NSSI group (*M* = 8.94, *SD* = 4.09) scored significantly higher than the comparison group (*M* = 4.13, *SD* = 3.28) on the persistence subscale [*t*(73) = −5.65, *p* < 0.001, Cohen’s *d* = 1.31]. Together, these results suggest individuals with a history of NSSI perceive themselves to be more sensitive to emotions, to feel emotions more strongly or intensely, and to experience these emotions for longer periods of time than those who do not engage in NSSI (see Tables 2, 3 for relevant means and standard deviations).

**Table 2:** Mean and standard deviations of trait emotion reactivity for NSSI group.

Emotion reactivity scale scores	Mean (standard deviation)
Total score	43.03 (18.86)
Sensitivity subscale score	18.83 (9.13)
Arousal subscale score	12.19 (5.84)
Persistence subscale	8.94 (4.09)

**Table 3:** Mean and standard deviations of trait emotion reactivity for comparison group.

Emotion reactivity scale scores	Mean (standard deviation)
Total score	20.85 (14.71)
Sensitivity subscale score	9.03 (7.33)
Arousal subscale score	5.77 (4.31)
Persistence subscale score	4.13 (3.28)

### State emotion reactivity

**Figure 1:**
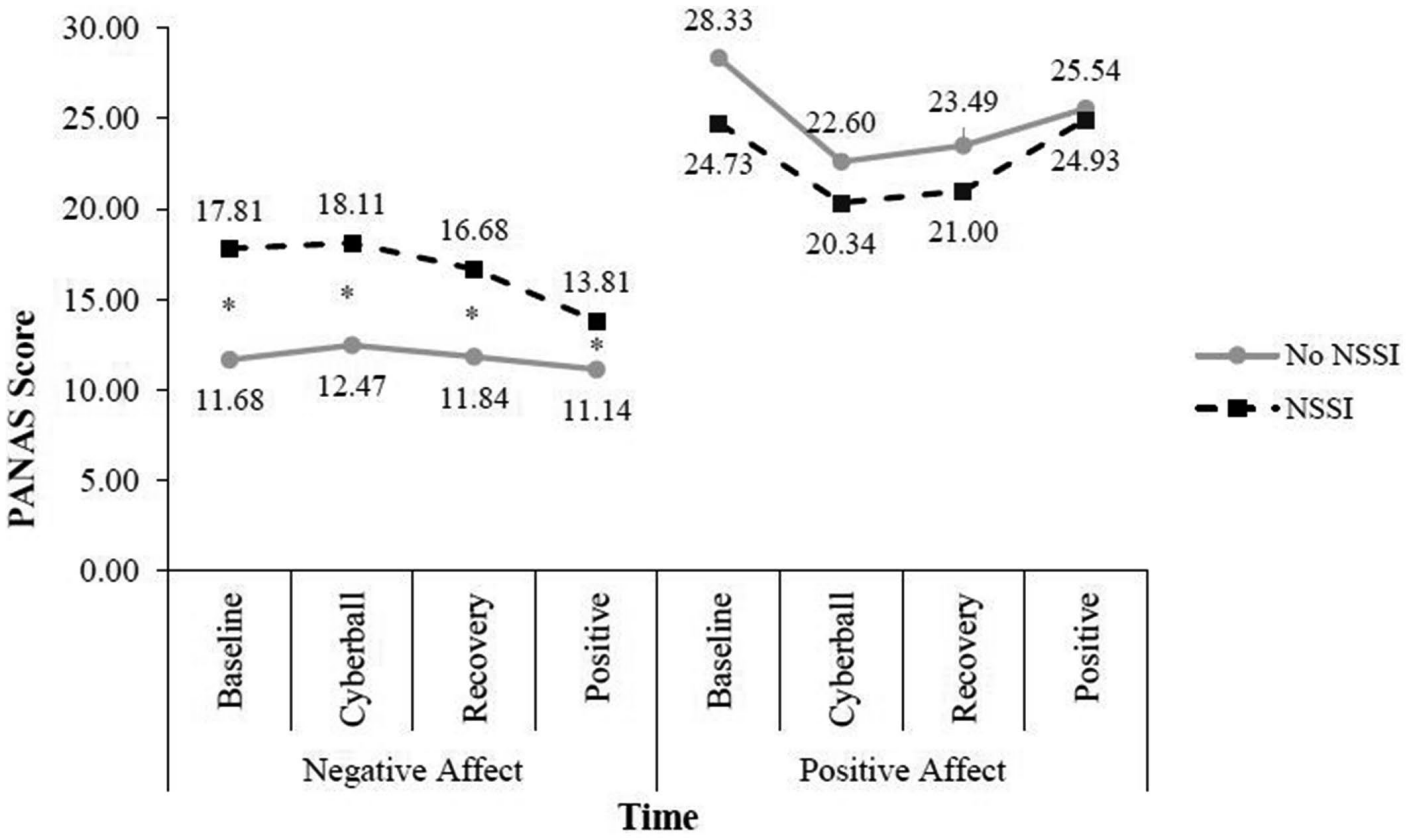
State emotion reactivity by NSSI group. *Indicates groups differ at the *p* < 0.05 level. No group differences in positive affect were observed.

Next, we examined whether there were any group differences in state emotion reactivity at any of the four time points on the positive/negative affect scales of the PANAS. We conducted two 4 (time: post-baseline video, post-Cyberball, post-recovery, post-positive mood) × 2 (group: NSSI history vs. no NSSI) repeated measures ANOVAs. For positive affect, Mauchly’s Test of Sphericity indicated the assumption of sphericity had been violated, *χ*^2^(5) = 23.40, *p* < 0.001, and a Greenhouse–Geisser correction was used. There was a significant main effect of time, *F*(3, 216) = 18.66, *p* < 0.001, *η*^2^ partial =0.206, indicating significant positive affect changes across events. Specifically, the total sample reported significantly less positive affect after Cyberball and recovery than they did following the neutral baseline video or the positive mood induction. There was no significant interaction between time and group (see Figure 1).

For negative affect, a Greenhouse–Geisser correction was again used [*χ*^2^(5) = 17.50, *p* = 0.004]. In this model, the main effect of time, *F*(3, 216) = 19.37, *p* < 0.001, *η*^2^ partial = 0.216, as well as the interaction of time by NSSI group were significant [*F*(3, 216) = 7.59, *p* < 0.001, *η*^2^ partial = 0.095]. Simple main effects showed that for the non-NSSI group negative affect after Cyberball and the positive mood induction were significantly different (*p* = 0.004) in that participants reported less negative affect following the positive mood induction, which suggests the positive mood induction facilitated mood repair following Cyberball. For the NSSI group, negative affect following the positive mood induction was significantly improved compared to the other three times (*p* < 0.001 for all). Overall, the NSSI group was significantly more negative at all-time points than the non-NSSI group (see Figure 1).

Given we were interested in emotion reactivity specifically in relation to a social stressor, we calculated change scores by subtracting post-Cyberball PANAS scores from baseline scores. When examining this reactivity, the groups did not significantly differ on the change in either positive, *t*(73) = −1.91, *p* = 0.238, or negative affect [*t*(51.35) = 0.408, *p* = 0.685]. Reactivity scores were also calculated for post-Cyberball to post-recovery and post-recovery to post-positive mood induction times. The only significant difference was post-recovery to post-positive mood induction in which the NSSI group experienced a greater reduction in negative affect [*t*(64.21) = 3.59, *p* = 0.001].

### Physiological reactivity

**Figure 2:**
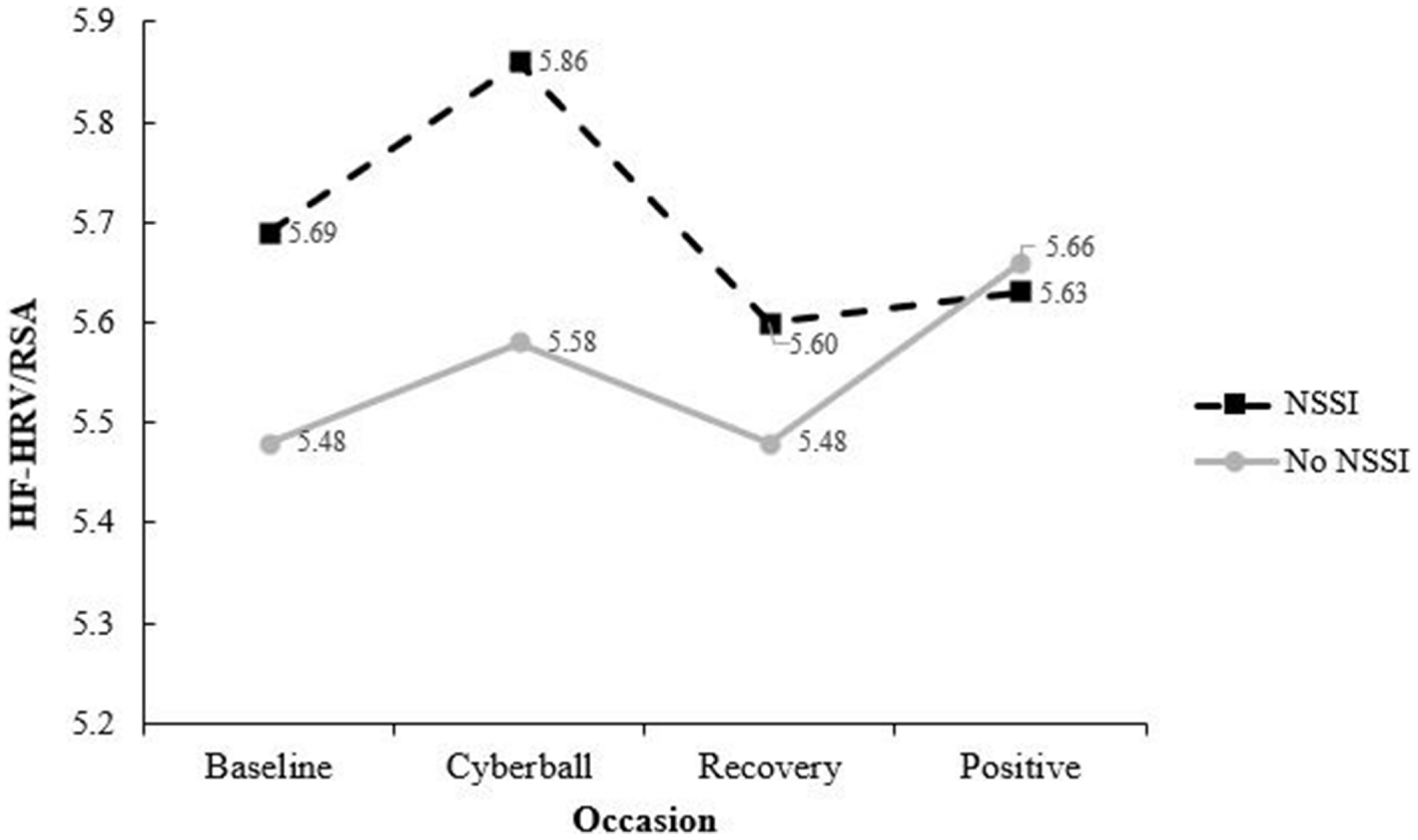
Physiological reactivity by NSSI group. NSSI, non-suicidal self-injury; groups were not significantly different at any timepoint, physiological reactivity was calculated by creating change scores by subtracting exclusion HF- HRV/RSA from baseline HF-HRV/RSA. The NSSI group was not significantly more reactive than the No NSSI group [*t*(58) = −0.45, *p* = 0.65].

To test whether NSSI history was related to HF-HRV/RSA, we conducted a 5 (time: baseline, inclusion, exclusion, recovery, positive mood) × 2 (group: NSSI history vs. no NSSI) repeated measures ANOVA. Mauchly’s test indicated assumptions of sphericity were violated and again a Greenhouse–Geisser correction was used. No significant main effect of time [*F*(3, 129.77) = 2.12, *p* = 0.104] or interaction [*F*(3, 129.77) = 1.66, *p* = 0.189] were found. As with state reactivity, change scores were calculated by subtracting post-Cyberball HF-HRV/RSA from baseline HF-HRV/RSA. Again, the NSSI group (*M* = 0.24, *SD* = 0.58) did not significantly differ from the non-NSSI group [(*M* = 0.13, *SD* = 0.62); *t*(59) = −0.73, *p* = 0.469]. As with state affect reactivity, this suggests individuals who engage in NSSI were not more physiologically reactive than individuals who do not. See Figure 2 for change score means and group patterns.

## Discussion

Emotion reactivity or the extent to which individuals are sensitive to and experience emotions intensely and persistently before returning to their normal level of arousal, is central to several theories of NSSI (e.g., Nock et al., 2008). Using a laboratory-based quasi-experimental design, we examined whether individuals with or without a history of NSSI differed on measures of trait, state, and physiological emotion reactivity in response to a social ostracism task, recovery period, and positive mood induction. As predicted, individuals with a history of NSSI endorsed greater levels of trait emotion reactivity than individuals without a history of NSSI. This finding is in line with prior cross-sectional studies that report group differences on trait measures of emotion reactivity (e.g., Nock et al., 2008; Glenn and Klonsky, 2011; Andover and Morris, 2014; Zelkowitz et al., 2016). These results confirm that individuals with a history of NSSI tend to globally appraise themselves to be more sensitive to emotions, to feel emotions more intensely, and to experience these emotions for longer periods of time than those who do not engage in NSSI.

Participants in the NSSI group reported greater negative affect at all-time points (i.e., baseline, post-Cyberball, recovery, and post-mood induction). There were no group differences in positive affect at any of the time points. The NSSI group did not exhibit greater emotion reactivity (i.e., greater increases in negative affect) in response to the social ostracism task or recovery period than the comparison group. Although this finding did not support our hypothesis, our results were consistent with some prior research, which found individuals with a history of NSSI did not exhibit greater emotion reactivity than the comparison group following an anger induction (Weinberg and Klonsky, 2012), stressful speech task (Franklin et al., 2010; Kaess et al., 2012), negative mood induction (Mettler et al., 2021) or in response to a social exclusion task (i.e., Cyberball; Schatten et al., 2015). It appears that individuals with NSSI history experience greater self-reported negative affect, but that this shift in reactivity does not translate into a significant change over time when compared to individuals without NSSI history.

In response to the positive mood induction, the NSSI group experienced greater mood repair and became significantly less sad than the non-NSSI group. This unexpected finding, if replicated, has potentially important clinical implications. For example, perhaps individuals with NSSI are particularly responsive to positive emotional experiences as evidenced by decreased negative affect. Despite its potential significance, research that experimentally induces positive mood is relatively scarce among samples with a history of NSSI. To our knowledge, only two other studies have examined emotion reactivity in response to a positive mood induction among individuals with or without a history of NSSI (Boyes et al., 2020; Mettler et al., 2021). Mettler et al. (2021) failed to find group differences in positive or negative emotion reactivity using the ERS (Nock et al., 2008) following a positive mood induction among a sample of women with a recent history of NSSI and a comparison group. Although Boyes et al. (2020) found individuals with a history of NSSI exhibited less positive affect in response to a positive mood induction, they did not assess changes in negative affect. It is possible that positive mood may subsequently reduce the risk of NSSI engagement by interrupting the maintenance cycle of NSSI (Hasking et al., 2018). If positive mood does function as a protective factor, effectively enhancing and regulating positive affect can be incorporated into the treatment of individuals with a history of NSSI to reduce the frequency and maintenance of this behavior.

The current study also examined whether individuals with or without a history of NSSI differed on a physiological measure (i.e., HF-HRV) of emotion reactivity in response to a social ostracism task. In contrast to predictions, groups did not differ on HF-HRV at baseline (i.e., resting) or in response to a social ostracism task (i.e., reactivity). Our results are counter to an earlier study that found adolescents with a history of self-injurious behaviors had decreased resting-state RSA and increased RSA withdrawal to a negative mood induction compared to those without a history of self-harm (Crowell et al., 2005); however, this study did not distinguish between suicide behaviors and non-suicidal self-injury. This raises a potential confound as prior work suggests a history of suicide attempts is associated with dysregulated HRV (Lin et al., 2015; Wilson et al., 2016). More recent work has failed to find group differences in resting-state HF-HRV among individuals with or without a history of NSSI (Giner-Bartolome et al., 2017; Koenig et al., 2017; Fox et al., 2018; Gratz et al., 2019). However, Fox et al. (2018) observed greater HF-HRV withdrawal following a 2 min social ostracism task among individuals with a history of recent NSSI compared to those without such a history. Our failure to find group differences might be due to the fact that we included participants who reported any lifetime history of NSSI, while Fox et al. (2018) restricted their NSSI group to those who engaged in NSSI within the past year. It is possible that more recent NSSI is associated with alterations in HF-HRV.

Our findings suggest regardless of NSSI history, the intensity and duration of negative affect in response to a social ostracism task is comparable and in line with Hooley and Franklin (2017), who argue the role of emotion reactivity is less central to NSSI. It is possible individuals with NSSI retrospectively recall or subjectively perceive their emotions as particularly intense and persistent due to a recall bias or prediction error, when their state emotional reactions are similar in strength and duration as those who do not engage in NSSI. This perception may reflect a tendency for individuals who engage in NSSI to appraise their emotions in response to a negative emotional experience in an overly negative manner. This would explain the differences found in trait emotion reactivity (i.e., total and subscale scores on the ERS) and the lack of group differences in subjective (i.e., change scores using the PANAS) and objective (i.e., HF-HRV) measures of emotion reactivity. According to Mennin and Fresco’s (2010) conceptual framework for understanding the relationship between emotion regulation and emotional disturbance, individuals with a range of psychopathology may react negatively toward their emotions. Negative reactivity can take the form of metacognitive beliefs, such as “*It is awful to experience negative emotions,*” and is theorized to contribute to more depression and anxiety symptoms and avoidant emotion regulation strategies (e.g., suppression). Indeed, recent research suggests that individuals with emotional disorders endorse more irrational beliefs about their emotions, which in turn were associated with more negative emotions and a lower perceived emotional control (Predatu et al., 2020).

While individuals with or without a history of NSSI may have similar levels of emotion reactivity, it is possible that similar levels of emotion reactivity lead to different outcomes depending on one’s history of NSSI. Individuals with a history of NSSI may be particularly sensitive to a normative increase in negative affect in response to negative stimuli or stress. Perhaps, similar to the stress sensitization literature (*cf.* Monroe and Harkness, 2005), over time normative levels of distress become capable of triggering NSSI behavior, or the level of distress required to precipitate NSSI decreases over time. Another possibility, in line with theories, suggests that NSSI is subject to both positive and negative reinforcement (Liu et al., 2016). More research is needed to examine within-person associations between affective states and NSSI, particularly how this relationship may be impacted by stress exposure and change over time (i.e., Miller et al., 2019). Given these possible idiographic differences in emotion reactivity among individuals, it may be important for clinicians to tailor interventions to individuals seeking ways to appraise and manage their emotion reactivity. These interventions could include DBT skills that focus on distress tolerance or emotion regulation skills, such as the STOP or TIP skills, or CBT treatments, like addressing cognitive distortions, to promote self-efficacy and effective coping.

### Limitations and future directions

Findings from the current study should be interpreted with the following limitations in mind. Our sample consisted of an ethnically and racially homogeneous sample of largely college students from the New England area of the United States. Though college students endorse rates of NSSI, our findings may not generalize to more diverse groups or to clinical populations. Additionally, we did not directly assess participants’ perception of exclusion on Cyberball. We used the VAS to calculate change in mood along a sad-to-happy continuum. Although the entire sample reported a statistically significant change in mood and increased sadness, it is unclear whether this shift is in response to feeling socially ostracized or to some other aspect of the task. Lastly, emotion reactivity and our ostracism task were tested in a controlled setting. In addition to laboratory-based assessments, future studies should include ecological momentary assessment to assess the impact of proximal processes (i.e., change in affect in response to social ostracism) in naturalistic settings over time.

## Conclusion

Individuals with a history of NSSI reported greater trait emotion reactivity but not greater emotional or physiological reactivity in response to a social ostracism task. This challenges traditional theories which suggest that individuals who engage in NSSI experience particularly intense and prolonged negative emotions in response to negative stimuli. While trait emotion reactivity may indirectly impact other processes associated with NSSI, such as a sense of self-efficacy or perceived control, a potential implication of these findings is that individuals who engage in NSSI may benefit from interventions that examine their appraisals about how they typically respond to distressing stimuli. In response to the positive mood induction, individuals with a history of NSSI demonstrated greater mood repair than the comparison group. If replicated, this finding may have clinical relevance.

## Data availability statement

The raw data supporting the conclusions of this article will be made available by the authors, without undue reservation.

## Ethics statement

The studies involving humans were approved by the University of Maine Institutional Review Board for the Protection of Human Subjects (IRB). The studies were conducted in accordance with the local legislation and institutional requirements. The participants provided their written informed consent to participate in this study.

## Author contributions

MJ: Writing – original draft, Writing – review & editing. AL: Writing – original draft, Writing – review & editing. VM: Writing – review & editing. MD: Writing – review & editing. EH: Writing – original draft, Writing – review & editing.
